# Development of an Improved Thiosulfate-Utilizing Denitrifying Bacteria-Based Ecotoxicity Test with High Detection Sensitivity and Reproducibility

**DOI:** 10.3390/toxics12110788

**Published:** 2024-10-29

**Authors:** Heonseop Eom

**Affiliations:** Department of Civil Engineering, Keimyung University, 1095 Dalgubeol-daero, Dalseo-gu, Daegu 42601, Republic of Korea; heom@kmu.ac.kr; Tel.: +82-53-580-5706

**Keywords:** microbial ecotoxicity, thiosulfate-utilizing denitrifying bacteria, detection sensitivity, test reproducibility

## Abstract

Microorganism-based ecotoxicity assessment has been widely used as a reliable tool showing direct biochemical impacts of contaminants on ecosystems and the environment. The present study aimed at developing a thiosulfate-utilizing denitrifying bacteria (TUDB)-based ecotoxicity test with high detection sensitivity and favorable reproducibility. To achieve this goal, existing TUDB toxicity tests were improved by employing a pure culture of *Thiobacillus thioparus* ATCC 8158 and optimizing test conditions, particularly in terms of inoculated microbial biomass, incubating temperature, and operational pH. From control tests, it was found that 4 h is a sufficient processing time for TUDB test kits. As a result of optimization, 20 mg VSS/L of initial bacterial biomass, 25 °C of incubating temperature, and 6 of operational pH were determined as the most favorable test conditions, providing enhanced detection sensitivity and reproducibility. Under these optimal test conditions, I conducted toxicity tests for diverse toxic metals and obtained 0.65 ± 0.03, 1.09 ± 0.04, 1.21 ± 0.07, 0.13 ± 0.01, 0.56 ± 0.04, 1.42 ± 0.03, 0.98 ± 0.02, and 2.12 ± 0.05 mg/L of 4 h EC_50_ values for Ag^+^, As^3+^, Cd^2+^, Cr^6+^, Cu^2+^, Hg^2+^, Ni^2+^, and Pb^2+^, respectively. These EC_50_ values are substantially lower than those from earlier TUDB tests, demonstrating the high detection sensitivity of the current TUDB tests. Moreover, the present TUDB tests attained very low coefficient of variation (CV) values (1.6–6.3%) for the EC_50_, showing favorable reproducibility of the test methodology. In addition, the current TUDB toxicity tests offer numerous advantages for ecotoxicity assessment, including versatility for diverse test samples, no requirement for advanced equipment, and no distortion of end-point measurement. These refinements render the TUDB tests a favorable ecotoxicity assessment with enhanced sensitivity and reproducibility.

## 1. Introduction

Ecotoxicity assessment is the process of evaluating potential adverse effects of contaminants or environmental stressors on ecosystems or the environment [1,2]. This process involves direct exposure of test organisms to pollutants and subsequent analysis of their biological response to the contaminants [3,4,5,6]. Because of such test methodology, ecotoxicity assessment better reveals the actual biochemical impacts of pollutants on ecological systems compared to conventional physicochemical quantity-based toxicity evaluation [7,8]. Moreover, ecotoxicity tests have straightforward experimental protocols and short processing time and do not generally require sophisticated analytical instruments or skilled personnel [7,8,9]. These advantages of ecotoxicity assessment make it a valuable alternative or supplement to physicochemical toxicity analysis [10,11,12].

In ecotoxicity tests, diverse trophic levels of living organisms ranging from higher organisms, such as fish and invertebrates, to water fleas and microorganisms have been employed [5,13,14,15,16]. Among these various organisms, microorganism-based tests are particularly cost-effective, provide high sensitivity and reproducibility, and represent less ethical concerns [8,17,18,19,20]. Numerous microorganisms, including microalgae, protozoa, fungi, and bacteria, have been used in ecotoxicity analysis and have shown favorable test performance in assessing diverse inorganic and organic contaminants [8].

Recently, Ashun et al. (2022) demonstrated the application of sulfur-oxidizing denitrifying bacteria in ecotoxicity assessments [21]. Specifically, they employed a mixed culture of thiosulfate-utilizing denitrifying bacteria (TUDB) as the test organisms. TUDB utilize thiosulfate (S_2_O_3_^2−^) and nitrate (NO_3_^−^) as electron donor and acceptor, respectively, and produce N_2_ gas as a result of their denitrifying metabolism [21,22,23] (Equation (1)). In the presence of contaminants, microbial activity of TUDB is limited and less N_2_ gas results. Hence, the toxicity of test samples is evaluated by comparing volumes of gas production (which can be easily measured by a glass syringe) between the test samples and controls (where no contaminants exist).

(1) $$5S_{2}O_{3}^{2-}+8NO_{3}^{-}+H_{2}O\to 4N_{2}+8SO_{4}^{2-}+2H^{+}$$

Because TUDB exist in diverse environmental spheres and play important roles in the nutrient cycling of sulfur and nitrogen, TUDB-based assessment appropriately demonstrates the toxicological impact of pollutants on the environment and ecology. Furthermore, the TUDB test can provide ecotoxicological information with no distortion. For example, test results from other microbial toxicity assessments employing light absorbance and color as end-point measurements can be distorted by conditions of test samples if test samples have high turbidity and specific color. However, the TUDB-based evaluation is not affected by these distortions. In addition, the TUDB test has a relatively simple test procedure and does not require advanced analytical instruments. Consequently, the TUBD-based assessment represents a reliable tool providing actual ecotoxicological information on contaminants. Ashun et al. (2022, 2023) confirmed that the TUDB test with measurement of gas production achieves favorable performance for toxicity assessment of metal-contaminated waters and soils [21,24].

The objective of the current study is to improve an existing TUDB toxicity test to obtain enhanced detection sensitivity and reproducibility. To achieve this goal, a pure culture of TUDB, specifically *Thiobacillus thioparus* ATCC 8158, was employed as the test organism. It was expected that this pure culture employment would respond to pollutants more sensitively and rapidly than a mixed culture of TUDB. In addition, test conditions in terms of inoculated bacterial biomass, incubating temperature, and operational pH were optimized. Through these optimizations, the current TUDB toxicity test attained improved sensitivity and reproducibility while decreasing processing time to 4 h.

## 2. Materials and Methods

### 2.1. TUDB Strain and Cultivation

This study used a pure culture of TUDB, specifically *Thiobacillus thioparus* ATCC 8158, as the test organism. This TUDB was obtained from the Microbial bioprocessing Laboratory at Yeungnam University (Gyeongsan, Republic of Korea). I cultured it using a liquid medium in a 1 L glass bottle connected to a gas bag to collect N_2_ gas generated from denitrification by TUDB. The liquid medium was prepared according to Chung et al. (2014) [22], and its pH was adjusted to 7 using MOPS (3-(N-morpholino)propanesulfonic acid) buffer (Sigma-Aldrich, St. Louis, MO, USA). As S and N sources for TUDB, thiosulfate (S_2_O_3_^2−^) and nitrate (NO_3_^−^) were employed, respectively, whose initial concentrations in the cultivation bottle were 7500 mg S/L and 500 mg N/L, respectively (S/N = 5). The medium was autoclaved for 2 h at 120 °C, then cooled to room temperature before use. A 5 mL filter-sterilized trace element solution, which was made according to Oh et al. (2011) [8], was added to the 1 L liquid medium. Prior to inoculation of TUDB, the liquid medium was sparged with 100% purity N_2_ gas (Daejeong, Daegu, Republic of Korea) for 3 h to create anaerobic conditions. Cultivation proceeded in an anaerobic shaking incubator (Mido, Busan, Republic of Korea) at 35 °C with 100 rpm. All glass bottles and parts were autoclaved before usage. I collected samples from the cultivation bottle and measured volatile suspended solids (VSS) daily to evaluate microbial growth. Generally, 3–4 days after starting cultivation, growth of TUDB reached the exponential growth phase, and I used the TUDB in this phase for subsequent control, optimization, and toxicity tests.

### 2.2. TUDB Toxicity Kit Tests

**Figure 1:**
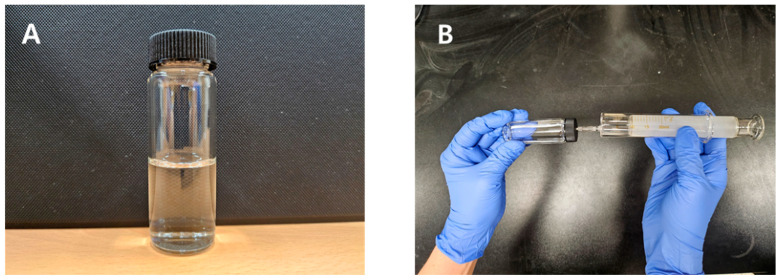
(**A**) A TUDB toxicity test kit. (**B**) Syringe method for measuring volume of produced gas from a test kit [25].

The TUDB test kit was developed based on a 25 mL glass vial with a rubber stopper and a plastic cap (Figure 1A, Duksan pure chemicals, Ansan, Republic of Korea). All glass vials were acid-washed, and all parts were autoclaved to prevent any microbial contamination. The TUDB kit consisted of a liquid medium (8 mL, identical to that used in the cultivation bottle) and TUDB (different amounts of TUDB depending on test purposes). The liquid medium was sparged with 100% purity N_2_ gas (Daejeong, Daegu, Republic of Korea) for 3 h before it was injected into the test kits, and test contaminants (toxic metals) were spiked if the TUDB kits were used for (metal) toxicity assessment. Initial concentrations of thiosulfate (S_2_O_3_^2−^) and nitrate (NO_3_^−^) in the test kits were 1500 mg S/L and 300 mg N/L, respectively. For experimental quality control, I measured S and N concentrations in the test kits using commercially available quantity analyzing kits (such as HS-S (Humas, Daejeon, Republic of Korea) for S and TNT836 (Hach, Loveland, CO, USA) for N) and the actual S and N concentrations in the test kits were 1499.7 mg S/L and 300.1 mg N/L, respectively. These results show that the nominal concentrations of S and N in the test kits are almost identical to the actual concentrations. The TUDB harvested from the cultivation bottle were employed as the test organisms in kit tests. After injecting the liquid medium and inoculating TUDB into the test kit, the glass vial was sealed with a rubber stopper and closed with a plastic cap. The rubber stopper was pierced by a needle of a syringe to release residual pressure. The TUDB kits were prepared in an anaerobic chamber. The TUDB kit tests involved incubation of the kits under specific temperatures for 4 or 5 h with 100 rpm shaking and measurement of volumes of produced gas from the test kits (after incubation) using a glass syringe (a syringe method measuring produced gas from the test kits is explained below). All kit tests were performed in triplicate, and the average values are presented in the results.

The TUDB test kits were employed in three experiments: (1) control tests, (2) optimization tests, and (3) metal toxicity tests. Test conditions of the TUDB kits varied depending on the test purposes. First, in the control tests, N_2_ gas production by TUDB was evaluated under different inoculated bacterial biomass (10, 20, 30, and 40 VSS mg/L), incubating temperatures (20, 25, 30, and 35 °C), and operational pH (5, 6, 7, and 8). Specifically, a total of 64 (4 × 4 × 4) combinations of test conditions were investigated. During 5 h of incubation, volumes of produced gas from the test kits were measured every 20 min (using a glass syringe). Based on the results, I developed gas production profiles showing accumulated volumes of produced gas and analyzed the times for completion of gas production under diverse test conditions.

The optimization tests were performed to determine test conditions providing high detection sensitivity and test reproducibility. Similar to the control tests, the optimization tests were also conducted under 64 test conditions. As the reference contaminant, different doses of Hg^2+^ (0.5, 1, 3, 5, and 10 mg/L) were spiked. In the optimization tests, we measured volumes of produced gas from the test kits after 4 h incubation (using a glass syringe). The optimization tests were performed in triplicate. Based on the average volume data, I analyzed the inhibition of TUDB (%) at each spiked dose and developed dose–response curves under the test conditions. Detection sensitivity and test reproducibility were evaluated by EC_50_ and coefficient of variation (CV) values for EC_50_, respectively. Considering the EC_50_ and CV results, the most favorable test conditions for the TUDB kits were determined.

Metal toxicity tests were conducted to assess the performance of the current TUDB test kits, and the results were compared to findings from an earlier study (Ashun et al., 2022) [21]. Tested toxic metals were Ag^+^, As^3+^, Cd^2+^, Cr^6+^, Cu^2+^, Hg^2+^, Ni^2+^, and Pb^2+^. I prepared metal stock solutions whose concentrations were 200 mg/L for all test metals. I took some amounts from these metal stock solutions and spiked them into the test kits to match nominal test concentrations. For experimental quality control, I measured actual metal concentrations in the stock solutions using a commercially available metal quantity analyzing kit (Humas, Daejeon, Republic of Korea; Hach, Loveland, CO, USA). As a result, actual metal concentrations of Ag^+^, As^3+^, Cd^2+^, Cr^6+^, Cu^2+^, Hg^2+^, Ni^2+^, and Pb^2+^ in their stock solutions were 200.0, 199.8, 199.7, 199.8, 200.0, 200.0, 200.1, and 199.9 mg/L, respectively, showing that the nominal metal concentrations in stock solutions were almost identical to the actual metal concentrations. Metal toxicity tests were conducted in triplicate under the optimal test conditions identified from the above optimization tests. After 4 h incubation, I measured volumes of generated gas (using a glass syringe) and analyzed EC_50_ and CV values from dose-response curves.

### 2.3. Syringe Method for Measuring Gas Production from TUDB Toxicity Kit

Volumes of produced gas from the TUDB test kits were measured using a 20 mL glass syringe (Figure 1B). Details of the measurement of gas production were based on the procedures from Eom et al. (2020) [25] and Ashun et al. (2023) [24]. First, a glass syringe (Hanmi, Suwon, Republic of Korea) plunger was lubricated using ionized water with 2–3 drops of detergent to facilitate its movement. For measurement of gas production, the glass syringe’s needle was inserted into the kit through the rubber stopper parallel to the ground. The syringe plunger was then allowed to move to reach an equilibrium between the gaseous phase of the test kit and atmospheric pressure. The displacement of the syringe plunger represents the volume of generated gas from the test kit.

### 2.4. Chemicals and Laboratory Analyses

All chemicals used in the current study were ACS grade, requiring no further purification. For S_2_O_3_^2−^ (S source) and NO_3_^−^ (N source), NaS_2_O_2_∙5H_2_O and KNO_3_ (Sigma-Aldrich, St. Louis, MO, USA) were used, respectively. For the metal toxicity tests, AgCl, NaAsO_2_, CdCl_2_, K_2_Cr_2_O_7_, CuSO_4_, HgCl_2_, NiCl_2_, and Pb(NO_3_)_2_ (Sigma-Aldrich, St. Louis, MO, USA) were employed. Bacterial biomass of inoculated TUDB was determined by VSS concentration, which was analyzed according to Standard Methods 2040D and E [26]. Operational pH in the TUDB test kits was adjusted using MOPS (3-(N-morpholino)propanesulfonic acid) buffer (Sigma-Aldrich, St. Louis, MO, USA).

Toxicity of contaminants was assessed by inhibition of TUDB (%). As Equation (2) describes, the inhibition of TUDB was determined by a comparison of volumes of produced gas between the controls (where no contaminant was spiked) and test samples.

(2) $$\mathrm{Inhibition}\;(\%)=(1-\frac{Volume\;of\;N2\;gas\;production\;from\;test\;sample\;for\;4\;h\;incubation}{Volum\;of\;N2\;gas\;productoin\;from\;control\;for\;4\;h\;incubation})\times 100$$

Based on inhibition data depending on the doses of spiked contaminants, dose–response curves were developed, and EC_50_ values for contaminants were determined from the Hillslope equation (Equation (3)).

(3) $$Y=Bottom+\left(\frac{Top-Bottom}{{\left(1+10\right)}^{\left((log{EC}_{50}-X)\times Hillslope\right)}}\right)$$

where X is the amount of contaminant, Y is the toxic response of TUDB (TUDB inhibition), Top is the maximum toxic response, and Bottom is the minimum toxic response.

CV values were determined by the ratios of the standard deviations to the mean values. To assess statistical significance among the data, the student *t*-test was performed. A *p*-value of less than 0.05 represents statistical significance.

## 3. Results

### 3.1. Control Tests

**Figure 2:**
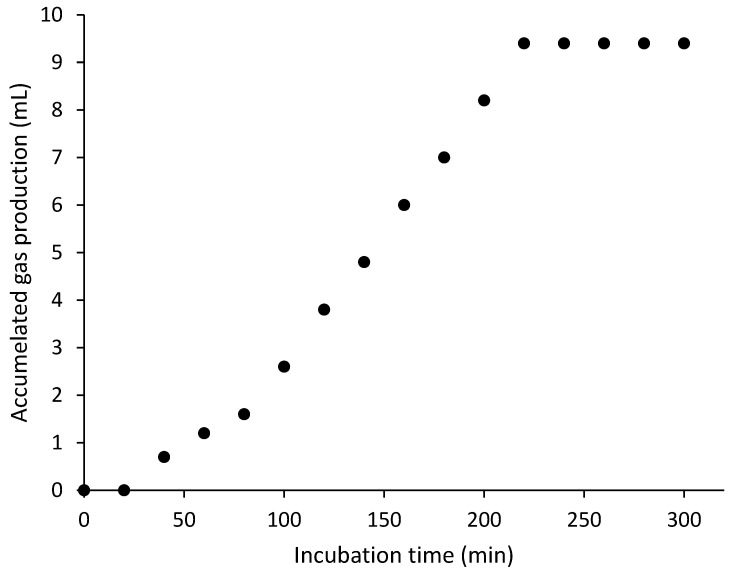
Gas production profile from one control kit test when inoculated bacterial biomass was 10 mg VSS/L, incubating temperature was 30 °C, and operational pH was 8.

To evaluate gas production by TUDB in the test kits, control tests were conducted under diverse test conditions. Figure 2 demonstrates one example of a gas production profile when inoculated bacterial biomass was 10 mg VSS/L, incubating temperature was 30 °C, and operational pH was 8. Under these test conditions, there was a lag phase for the first 20 min, then gas production continued for 3 h and 20 min until the maximum gas production (9.4 mL) was achieved.

Data of total volumes of produced gas and times for completion of gas generation from the control tests are summarized in Table 1. The amounts of produced gas were largely comparable (9.2–9.6 mL) regardless of test conditions. Because identical amounts of S and N were employed in all test kits, the total volumes of produced gas by TUDB were similar. However, the times to attain the maximum gas production varied (2 h and 40 min–3 h and 40 min) depending on the test conditions. For example, when inoculated bacterial biomass was 40 mg VSS/L, incubating temperature was 35 °C, and operational pH was 7, the time for completing gas production was 2 h and 40 min. However, when inoculated bacterial biomass was 10 mg VSS/L, incubating temperature was 20 °C, and operational pH was 5, the time was 3 h and 40 min. In general, as the inoculated bacterial biomass was greater, the incubating temperature was higher, and the operational pH approximated 7, the times for completion of gas production by TUDB were shorter. I speculate that these time results can serve as a proxy for the microbial activity of TUDB in test kits. If the test conditions are favorable for TUDB, the time taken to complete gas generation was short. If unfavorable, the time was long. In addition, because the longest time for completion of gas production was 3 h and 40 min, I determined that 4 h is an appropriate incubating time for TUDB toxicity test processing.

### 3.2. Optimization Tests

**Figure 3:**
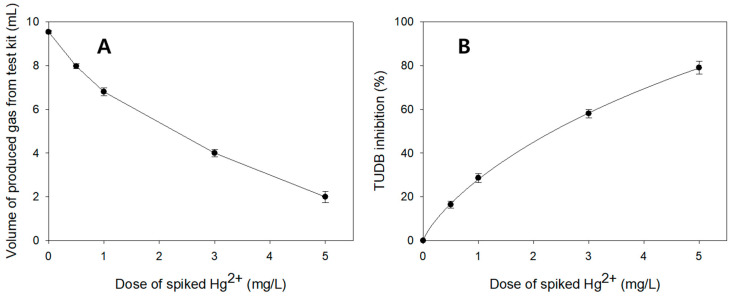
Results from one optimization kit test when inoculated microbial biomass was 20 VSS mg/L, incubating temperature was 20 °C, and operation pH was 8. (**A**) Volumes of produced gas from test kits depending on doses of spiked Hg^2+^. (**B**) Dose–response curve (doses of spiked Hg^2+^ VS. % TUDB inhibition). Symbols and error bars represent the average values and standard deviations from the triplicate tests.

To improve detection sensitivity and reproducibility of the TUDB toxicity tests, optimization for test conditions was performed, particularly as regards inoculated bacterial biomass, incubating temperature, and operational pH. Figure 3A illustrates results (volumes of produced gas from test kits depending on doses of spiked Hg^2+^) from one optimization test when inoculated microbial biomass was 20 VSS mg/L, incubating temperature was 20 °C, and operation pH was 8. As greater doses of Hg^2+^ were introduced into the test kits, the volumes of produced gas decreased. For example, when spiked Hg^2+^ concentrations were 0, 0.5, 1, 3, and 5 mg/L, the average volumes of gas generated from the test kits (after 4 h incubation) were 9.5 ± 0.1, 8.0 ± 0.1, 6.8 ± 0.2, 4.0 ± 0.2, and 2.0 ± 0.3 mL, respectively. Based on these data, a dose–response (% inhibition) curve was developed in Figure 3B. Under the above test conditions, the 4 h EC_50_ value for Hg^2+^ was 2.36 ± 0.14 mg/L, and the CV value for the EC_50_ was 6.0%.

Table 2 summarizes the 4 h EC_50_ and CV values from all kit tests conducted for optimization of test conditions. First, in terms of inoculated bacterial biomass, employment of 10 or 20 mg VSS/L biomass led to lower EC_50_ values than inoculation of 30 or 40 mg VSS/L biomass if other test conditions (incubating temperature and operational pH) were identical. For example, when 10, 20, 30, and 40 mg VSS/L bacterial biomass were employed under 25 °C of incubating temperature and operational pH of 6, 1.40 ± 0.34, 1.42 ± 0.03, 2.80 ± 0.06, and 3.17 ± 0.09 mg/L of EC_50_ values resulted, respectively. Between 10 and 20 mg VSS/L initial biomass, 10 mg VSS/L initial biomass led to slightly lower EC_50_ values than 20 mg VSS/L initial biomass. However, 10 mg VSS/L initial biomass resulted in substantially greater CV values for the EC_50_ compared to 20, 30, or 40 mg VSS/L initial biomass under all tested temperatures and pH. Specifically, inoculation of more than 20 mg VSS/L bacterial biomass led to 1.4–6.0% of CV values (except when pH was 5), whereas employment of 10 mg VSS/L bacterial biomass caused 20.2–26.3% of CV values (except when pH was 5). These data suggest that inoculation of 10 mg VSS/L bacterial biomass yielded significantly worse test reproducibility than employment of 20, 30, and 40 mg VSS/L bacterial biomass. These findings imply that initial microbial biomass affects both detection sensitivity and test reproducibility. Hence, it is necessary to identify adequate initial bacterial biomass that balances sensitivity and reproducibility. Based on the above EC_50_ and CV values, I determined that 20 mg VSS/L is the optimal initial bacterial biomass to achieve enhanced sensitivity and reproducibility in the TUDB tests.

With respect to incubating temperature, the TUDB kits tested at 20 and 25 °C resulted in better detection sensitivity than those at 30 and 35 °C if initial bacterial biomass and operational pH were identical. For example, the TUDB tests conducted at 20, 25, 30, and 35 °C with 20 mg VSS/L of initial bacterial biomass and operational pH of 6 resulted in 1.98 ± 0.08, 1.42 ± 0.03, 2.61 ± 0.07, and 2.81 ± 0.11 mg/L of (4 h) EC_50_ values for Hg^2+^, respectively. These results indicate that relatively lower incubating temperatures can enhance detection sensitivity in the current TUDB tests. However, test reproducibility was not influenced by incubating temperatures if the other test conditions were the same. For example, the above kits (20 mg VSS/L of initial bacterial biomass and 6 of operational pH) at 20, 25, 30, and 35 °C of incubating temperatures showed 2.2–4.0% of CV values for the EC_50_, whose differences were not statistically significant. Since 25 °C provided better sensitivity and reproducibility than 20 °C, I chose 25 °C as the optimal incubating temperature for the present TUDB kit tests.

As a result of optimization tests conducted under diverse pH, pH 6 presented the lowest EC_50_ values compared to pH 5, 7, and 8 under all tested initial biomass and incubating temperatures. For example, the EC_50_ values from the tests with pH 5, 6, 7, and 8 under 20 mg VSS/L of initial microbial biomass and 30 °C of incubating temperature were 3.11 ± 0.44, 2.61 ± 0.07, 2.85 ± 0.09, and 3.04 ± 0.12 mg/L, respectively. These data support that pH 6 is the optimal pH condition, allowing improved detection sensitivity. However, the CV values were largely similar when operational pH values were 6, 7, and 8, although the tests with pH 5 showed particularly high CV values. These results indicate that pH is not a significant factor affecting test reproducibility unless pH is quite acidic, such as pH 5. Considering these EC_50_ and CV values, pH 6 is the favorable pH condition for improved detection sensitivity and test reproducibility. In summary, 20 mg VSS/L of initial bacterial biomass, 25 °C of incubating temperature, and operational pH of 6 were the optimal test conditions, providing favorable test performance.

### 3.3. Comparisons of TUDB Toxicity Test Results Between the Present and Earlier Techniques

**Figure 4:**
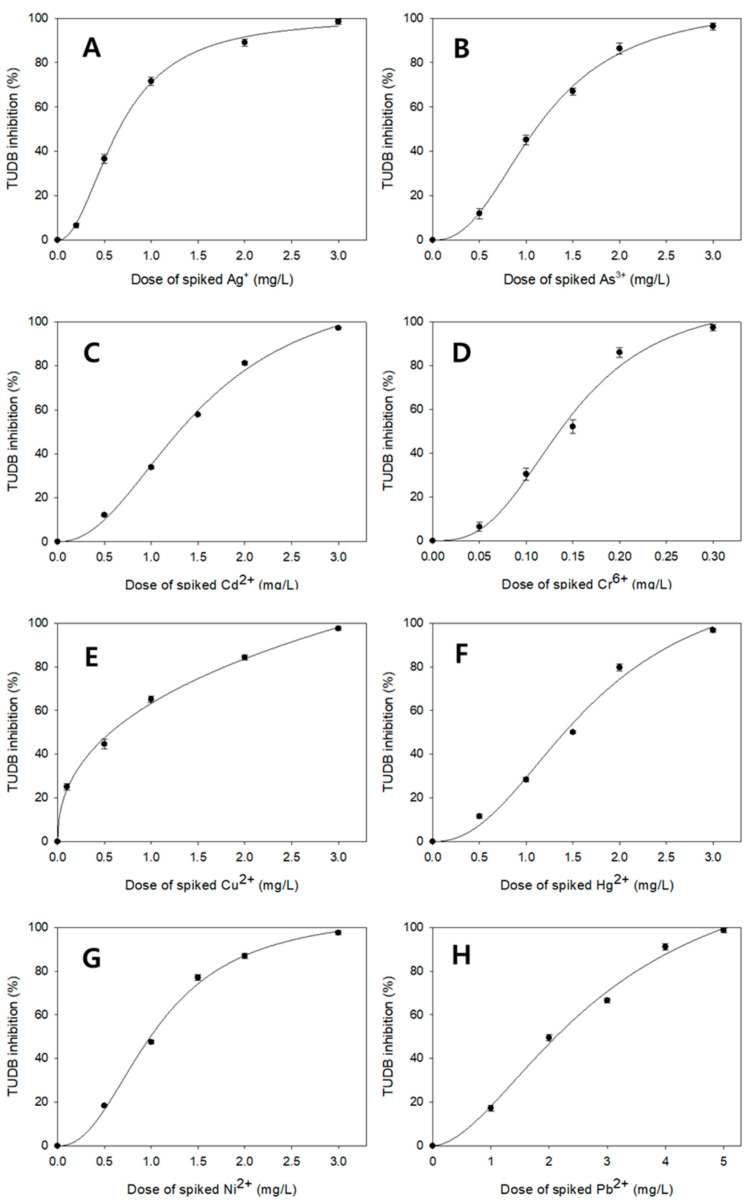
Dose-response curves (Dose of spiked toxic metal VS. % TUDB inhibition) from metal toxicity tests conducted under optimized test conditions. (**A**) Ag^+^, (**B**) As^3+^, (**C**) Cd^2+^, (**D**) Cr^6+^, (**E**) Cu^2+^, (**F**) Hg^2+^, (**G**) Ni^2+^, (**H**) Pb^2+^.

I performed toxicity assessment for diverse metals (Ag^+^, As^3+^, Cd^2+^, Cr^6+^, Cu^2+^, Hg^2+^, Ni^2+^, and Pb^2+^) using the present TUDB test kits under the above optimal test conditions. Figure 4 illustrates dose–response curves from the current metal toxicity tests. As a result, the current test technique led to significantly lower EC_50_ values for metals than those based on earlier test methodology. For example, Ashun et al. (2022) obtained 2.90, 4.10, 5.56, 0.51, 2.90, 8.06, 3.60, and 19.3 mg/L of 24 h EC_50_ data for Ag^+^, As^3+^, Cd^2+^, Cr^6+^, Cu^2+^, Hg^2+^, Ni^2+^, and Pb^2+^, respectively [21]. However, the present tests with optimal conditions resulted in 0.65 ± 0.03, 1.09 ± 0.04, 1.21 ± 0.07, 0.13 ± 0.01, 0.56 ± 0.04, 1.42 ± 0.03, 0.98 ± 0.02, and 2.12 ± 0.05 mg/L of 4 h EC_50_ values for Ag^+^, As^3+^, Cd^2+^, Cr^6+^, Cu^2+^, Hg^2+^, Ni^2+^, and Pb^2+^, respectively. This comparison indicates that the present optimized test technique achieved improved detection sensitivity for metal toxicity assessment. It is noteworthy that the current technique showed lower EC_50_ results even with a decreased incubating time (4 h). In the earlier study, 24 h incubating time was necessary. However, the current study significantly shortened the incubating time to 4 h. In addition, the current test technique attained substantially low CV values, 1.6–6.3%, for toxic metals, demonstrating favorable test reproducibility of the current TUDB test methodology.

## 4. Discussion

In the present study, I aimed to improve earlier TUDB tests to achieve better toxicity detection sensitivity and favorable reproducibility. Control tests were performed to investigate gas production by TUDB in test kits and evaluate the necessary incubating time for toxicity test processing. As discussed above, total volumes of produced gas from control test kits were largely similar (9.2–9.6 mL) regardless of test conditions. Because every test kit had identical amounts of S (electron donor) and N (electron acceptor), volumes of N_2_ gas produced from denitrification by TUDB were comparable. These observed volume data are similar to the findings from Ashun et al. (2023) [21,24]. The earlier studies also reported 9–10 mL of produced gas volume from control kit tests when their test and nutritional conditions were comparable to the present test. Although I did not directly analyze the type of gas produced from the kit tests during this study, my earlier preliminary study investigated gas produced from control kit tests (which were conducted under similar conditions to the current test) using gas chromatography (GC, Thermo Trace 1300GC, Thermo Fisher Scientific, Waltham, MA, USA) equipped with a Molecular Sieve 5A column (Thermo Fisher Scientific, Waltham, MA, USA) and a thermal conductivity detector (TCD, Thermo Fisher Scientific, Waltham, MA, USA) and found that the only gas detected by GC-TCD was nitrogen gas.

Times for the completion of gas production in control kit tests represent the microbial activity of TUDB in test kits. The shortest time (2 h and 40 min) (for gas production to be completed) was attained when 40 mg VSS/L bacterial biomass was inoculated, the incubating temperature was 30–35 °C, and operational pH was 6–7. Given that TUDB are mesophilic bacteria and can be highly active at a neutral pH condition [27,28,29], this result seems to be reasonable. Moreover, the longest time for completion of gas production from kit tests was 3.8 h. Accordingly, 4 h is a sufficient processing time for the present TUBD kit tests.

The first measure that I took to enhance the test performance of the TUDB tests was the employment of a pure culture of *Thiobacillus thioparus* ATCC 8158 as the test organism. In the earlier TUDB toxicity tests [21], the authors used a mixed culture comprising diverse TUDB such as *Thiobacillus thioparus*, *Thioprofundum hispidum*, *Sulfurovum denitrificans*, and *Acholeplasma granularum*. I estimate that a symbiotic relationship among various TUDB in a mixed culture may cause TUDB to be less inhibited by toxic substances. Furthermore, the toxic mechanism and impact of toxic metals vary depending on the species of TUDB, possibly leading to decreased inhibitory effects on the overall community of TUDB. Consequently, the TUDB kits with a mixed culture showed relatively low sensitivity to toxicants. However, we expected that the employment of a pure culture of TUDB could resolve this issue because one specific species of TUDB is more vulnerable to toxic substances. A study by Eom (2023) confirmed that inoculation of one specific species improves the detection sensitivity in microbial ecotoxicity assessment compared to the employment of a mixed culture composed of diverse microorganisms [20].

In addition, I optimized the test conditions of the TUDB test kit, particularly with respect to inoculated bacterial biomass, incubating temperature, and operational pH, to improve detection sensitivity and test reproducibility. The literature reports that initial bacterial biomass is a critical factor in determining detection sensitivity. Numerous microbial ecotoxicity studies, including Lin et al. (2005), Singh and Shrivastava (2015), Eom et al. (2021), and Eom (2023), have demonstrated that decreased initial microbial biomass leads to an improvement in detection sensitivity [20,30,31,32]. Because toxicant availability per cell is determined by amounts of cells, inoculation of less biomass can cause greater inhibition on test organisms and thus increase toxicity detection sensitivity. However, too low initial biomass can adversely affect test reproducibility. Lin et al. (2005), Eom et al. (2021), and Eom (2023) showed that if initial microbial biomass falls below a certain level, test reproducibility significantly deteriorates [20,30,32]. The current study also observed that test reproducibility became impaired when initial bacterial biomass was less than 20 mg VSS/L. Hence, it is necessary to determine an appropriate initial bacterial biomass that balances sensitivity and reproducibility. In this study, it was 20 mg VSS/L.

Incubating temperatures and operational pH were also important factors affecting detection sensitivity. In general, these factors are relevant to the microbial activity of test organisms. It was found that the test conditions creating TUDB high microbial activity did not necessarily provide enhanced detection sensitivity. For example, the test conditions attaining the shortest time for completion of gas production in the control tests did not lead to the lowest EC_50_ value in the optimization tests. This is most likely because test organisms with high activity are not readily inhibited by toxicants. Eom et al. (2021) and Eom (2023) likewise demonstrated that microbial toxicity tests do not result in favorable detection sensitivity when test organisms show high metabolic activity [20,32]. However, with regard to test reproducibility, incubating temperature and operational pH were not critical factors. Regardless of tested temperature and pH, the CV values for the EC_50_ from the optimization tests were largely in the range of 1.4–6.0% (except for a very low operational pH such as pH 5). Given earlier reports that conventional algal toxicity tests have 20–32% test variability in terms of reproducibility [32,33,34], my CV values indicate that the present TUDB tests have favorable test reproducibility.

In addition to enhanced detection sensitivity and favorable test reproducibility, the current TUDB test kits offer a number of advantages, such as ecotoxicity assessment. First, TUDB are chemolithoautotrophic bacteria, indicating that TUDB are not affected by organic substances in test samples. If test organisms are heterotrophic, their activity is substantially influenced by organic matters in test samples, leading to volatility in test results depending on the organic substances in test samples. As discussed in the introduction, the employment of gas production as an end-point measurement is another advantage of the current test. In many microbial toxicity tests, light absorbance and color are used as end-point measurements [9]. Values from these analyses are substantially affected by the conditions of test samples. If test samples have high turbidity and specific color, test results can be distorted. However, our end-point measurement is not affected by these distortions. Moreover, the current TUDB tests only need a glass syringe for the end-point measurement, whereas other microbial tests sometimes require advanced analytic instruments [9]. This advantage can make the TUDB toxicity test more economical.

In the present study, I optimized the test conditions of earlier TUDB tests and achieved improved detection sensitivity and favorable reproducibility with shorter processing times. My TUDB tests were developed based on portable kit-type bioassays, making my tests suitable for field application. However, for even better field applications, I still need a field-applicable incubating system. I am currently developing this portable system. Furthermore, my future research includes toxicity assessment for diverse inorganic and organic contaminants using my TUDB toxicity tests. Through these future studies, I will continue to evaluate the reliability of my TUDB tests.

## 5. Conclusions

The current study aimed to optimize an existing TUDB-based toxicity test to have better detection sensitivity and favorable reproduction. To attain this goal, I employed a pure culture of TUDB, *Thiobacillus thioparus* ATCC 8158. I hypothesized that the pure culture would show high responsiveness to contaminants compared to a mixture culture of TUDB. Moreover, I optimized the test conditions of the TUDB test kits in terms of inoculated microbial biomass, incubating temperature, and operational pH. From the results of control tests, I found that 4 h is adequate incubating time for toxicity test processing. Optimization tests demonstrated that 20 mg VSS/L of initial bacterial biomass, 25 °C of incubating temperature, and operational pH of 6 are the favorable test conditions providing high sensitivity and reproducibility. Under these optimal test conditions, I performed toxicity assessments for diverse toxic metals. As a result, the 4 h EC_50_ values for Ag^+^, As^3+^, Cd^2+^, Cr^6+^, Cu^2+^, Hg^2+^, Ni^2+^, and Pb^2+^ were 0.65 ± 0.03, 1.09 ± 0.04, 1.21 ± 0.07, 0.13 ± 0.01, 0.56 ± 0.04, 1.42 ± 0.03, 0.98 ± 0.02, and 2.12 ± 0.05 mg/L, respectively. These lower EC_50_ values demonstrate that the current optimized TUDB test technique attained better test performance compared to earlier TUDB test methodology. Furthermore, I obtained 1.6–6.3% CV values for the EC_50_ from metal toxicity tests, showing favorable test reproducibility of the present test method. In addition, the TUDB tests have numerous advantages as ecotoxicity tests, such as wider application for diverse test samples, no need for advanced analytical instruments, and avoiding distortion of test results originating from the characteristics of test samples.

## Figures and Tables

**Table 1:** Data of volumes of produced gas and times for completion of gas production from control kit tests.

Initial Bacterial Biomass (mg VSS/L)	Incubating Temperature (°C)	Operational pH	Volume of Total Produced Gas (mL)	Time for Completion of Gas Production	Initial Bacterial Biomass (mg VSS/L)	Incubating Temperature (°C)	Operational pH	Volume of Total Produced Gas (mL)	Time for Completion of Gas Production
10	20 °C	5	9.2	3 h 40 min	30	20 °C	5	9.4	3 h 20 min
		6	9.3	3 h 40 min			6	9.4	3 h 20 min
		7	9.4	3 h 20 min			7	9.6	3 h 00 min
		8	9.4	3 h 40 min			8	9.6	3 h 20 min
	25 °C	5	9.3	3 h 40 min		25 °C	5	9.4	3 h 20 min
		6	9.3	3 h 40 min			6	9.4	3 h 20 min
		7	9.4	3 h 20 min			7	9.6	3 h 00 min
		8	9.4	3 h 40 min			8	9.6	3 h 20 min
	30 °C	5	9.3	3 h 40 min		30 °C	5	9.4	3 h 20 min
		6	9.3	3 h 40 min			6	9.6	3 h 20 min
		7	9.5	3 h 20 min			7	9.6	3 h 00 min
		8	9.4	3 h 40 min			8	9.6	3 h 00 min
	35 °C	5	9.2	3 h 40 min		35 °C	5	9.4	3 h 20 min
		6	9.3	3 h 20 min			6	9.4	3 h 00 min
		7	9.5	3 h 0 min			7	9.6	2 h 40 min
		8	9.5	3 h 20 min			8	9.6	3 h 00 min
20	20 °C	5	9.4	3 h 40 min	40	20 °C	5	9.2	3 h 20 min
		6	9.3	3 h 40 min			6	9.3	3 h 20 min
		7	9.4	3 h 20 min			7	9.6	3 h 00 min
		8	9.5	3 h 40 min			8	9.4	3 h 00 min
	25 °C	5	9.4	3 h 40 min		25 °C	5	9.4	3 h 20 min
		6	9.4	3 h 40 min			6	9.5	3 h 20 min
		7	9.5	3 h 20 min			7	9.6	3 h 00 min
		8	9.5	3 h 40 min			8	9.4	3 h 20 min
	30 °C	5	9.3	3 h 20 min		30 °C	5	9.4	3 h 00 min
		6	9.4	3 h 20 min			6	9.5	3 h 00 min
		7	9.6	3 h 20 min			7	9.6	2 h 40 min
		8	9.5	3 h 40 min			8	9.6	2 h 40 min
	35 °C	5	9.2	3 h 20 min		35 °C	5	9.4	3 h 20 min
		6	9.4	3 h 20 min			6	9.6	3 h 00 min
		7	9.4	3 h 00 min			7	9.6	2 h 40 min
		8	9.5	3 h 20 min			8	9.5	2 h 40 min

**Table 2:** Data of 4 h EC_50_ values and CV for EC_50_ from optimization kit tests.

Initial Bacterial Biomass (mg VSS/L)	Incubating Temperature (°C)	Operational pH	4 h EC50 (mg/L)	CV (%)	Initial Bacterial Biomass (mg VSS/L)	Incubating Temperature (°C)	Operational pH	4 h EC50 (mg/L)	CV (%)
10	20 °C	5	2.66 ± 0.84	31.4	30	20 °C	5	4.05 ± 0.50	12.4
		6	1.67 ± 0.41	24.4			6	3.08 ± 0.08	2.6
		7	2.01 ± 0.41	20.4			7	3.64 ± 0.18	4.8
		8	2.13 ± 0.45	21.3			8	3.53 ± 0.20	5.6
	25 °C	5	1.71 ± 0.53	31.2		25 °C	5	3.41 ± 0.54	15.8
		6	1.44 ± 0.34	23.7			6	2.80 ± 0.06	2.1
		7	1.69 ± 0.40	23.5			7	3.02 ± 0.09	3.0
		8	1.68 ± 0.44	26.3			8	3.25 ± 0.09	2.8
	30 °C	5	3.06 ± 0.93	30.3		30 °C	5	4.15 ± 0.51	12.3
		6	2.42 ± 0.52	21.4			6	3.82 ± 0.21	5.5
		7	2.77 ± 0.59	21.4			7	4.28 ± 0.22	5.1
		8	3.02 ± 0.61	20.2			8	4.04 ± 0.06	1.4
	35 °C	5	3.17 ± 1.00	31.6		35 °C	5	4.28 ± 0.52	12.1
		6	2.67 ± 0.56	21.0			6	4.11 ± 0.09	2.2
		7	2.82 ± 0.59	21.0			7	4.32 ± 0.17	4.0
		8	2.97 ± 0.67	22.6			8	4.22 ± 0.20	4.7
20	20 °C	5	2.75 ± 0.44	16.1	40	20 °C	5	4.10 ± 0.46	11.3
		6	1.98 ± 0.08	4.0			6	3.65 ± 0.11	3.0
		7	2.26 ± 0.13	5.6			7	3.78 ± 0.18	4.8
		8	2.36 ± 0.14	6.0			8	3.99 ± 0.11	2.8
	25 °C	5	1.92 ± 0.27	14.1		25 °C	5	3.99 ± 0.44	11.1
		6	1.42 ± 0.03	2.2			6	3.17 ± 0.09	2.7
		7	1.83 ± 0.09	4.7			7	3.71 ± 0.10	2.7
		8	1.82 ± 0.06	3.3			8	3.94 ± 0.10	2.6
	30 °C	5	3.11 ± 0.44	14.0		30 °C	5	4.80 ± 0.48	10.1
		6	2.61 ± 0.07	2.8			6	4.21 ± 0.07	1.7
		7	2.85 ± 0.09	3.1			7	4.67 ± 0.09	1.9
		8	3.04 ± 0.12	4.0			8	4.62 ± 0.05	1.0
	35 °C	5	3.35 ± 0.53	15.9		35 °C	5	4.93 ± 0.70	14.3
		6	2.81 ± 0.11	3.8			6	4.55 ± 0.09	1.9
		7	3.07 ± 0.14	4.6			7	4.89 ± 0.08	1.5
		8	3.19 ± 0.13	4.1			8	4.94 ± 0.09	1.7

## Data Availability

The data presented in this study are available upon request from the corresponding author.
